# Clinical Presentation and Management of Patients Referred for Transthoracic Echocardiography in Canada’s Arctic Region

**DOI:** 10.1016/j.cjco.2026.03.007

**Published:** 2026-04-01

**Authors:** Gaspard Suc, Ian G. Burwash, Luc Beauchesne, Francois de Wet, Lawrence Lau, Hassan Alfraidi, Anahita Tavoosi, Andrew Mulloy, David Ian Paterson, Markus Schwerzmann, Hassan Mir, Ellamae Stadnick, Kwan L. Chan, Hanh Nguyen, Christele Ferry, Roja Gauda, Rob S. Beanlands, David H. Birnie, Mehrdad Golian, Richard F. Davies, David Messika-Zeitoun

**Affiliations:** a Division of Cardiology, University of Ottawa Heart Institute, Ottawa, Ontario, Canada; b Nunavut's Department of Health, Nunavut, Canada; c Division of Cardiac Prevention and Rehabilitation, University of Ottawa Heart Institute, Ottawa, Ontario, Canada; d Nunavut Community Cardiologist, Nunavut, Ontario, Canada; e School of Epidemiology and Public Health, Faculty of Medicine, University of Ottawa, Ottawa, Ontario, Canada

**Keywords:** transthoracic echocardiography, Indigenous, equity, valvular heart disease

## Abstract

**Background:**

Indigenous populations in Canada experience disproportionately high cardiovascular risk, yet contemporary data on echocardiographic findings in the Arctic remain limited. This study evaluated indications, echocardiographic findings, and subsequent management of patients undergoing transthoracic echocardiography in Eastern Nunavut.

**Methods:**

We conducted a retrospective analysis of all consecutive transthoracic echocardiograms performed between January 2020 and December 2023 in Iqaluit and surrounding remote communities. Demographic data, echocardiographic results, and management decisions were collected and compared between Indigenous and non-Indigenous patients. Significant abnormalities were defined as moderate or severe valvular heart disease (VHD), prosthetic valve dysfunction, left ventricular ejection fraction (LVEF) < 50%, regional wall-motion abnormalities (RWMA), systolic pulmonary artery pressure ≥ 40 mm Hg, or congenital heart disease.

**Results:**

Among 662 patients (mean age 55 ± 16 years; 47% women), 530 (80%) were Indigenous. Significant abnormalities were identified in 197 patients (30%). Moderate or severe VHD was present in 67 patients (10%), most commonly degenerative (31%), followed by functional (25%) and rheumatic (16%) etiologies. Reduced LVEF was seen in 16%, and RWMA was seen in 7%. Management included local follow-up (44%), transfer or hospital admission (16%), and tertiary-care referral (9%). Compared to non-Indigenous patients, Indigenous patients had higher unadjusted rates of significant abnormalities (33% vs 18%, *P* < 0.01), moderate or severe VHD (12% vs 5%, *P* = 0.04), and reduced LVEF or RWMA (21% vs 13%, *P* = 0.07).

**Conclusions:**

Patients in Canada’s Arctic, particularly Indigenous communities, carry a substantial burden of cardiac disease, including elevated rates of VHD and ventricular dysfunction. Improved prevention and access to cardiovascular care are essential to reduce these disparities.

## Central Illustration

**Figure:**
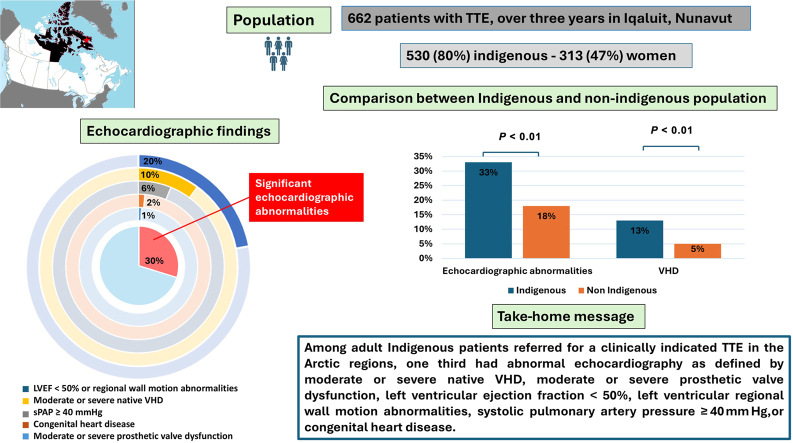

## Résumé

**Contexte:**

Les populations autochtones du Canada sont exposées à un risque cardiovasculaire significativement plus élevé, mais les données contemporaines sur les résultats des examens échocardiographiques réalisés dans l’Arctique demeurent limitées. Cette étude visait à évaluer les indications et les résultats des examens d’échocardiographie transthoracique (ETT) ainsi que la prise en charge subséquente des patients qui se prêtent à cette intervention dans l’Est du Nunavut.

**Méthodologie:**

Nous avons procédé à l’analyse rétrospective de toutes les ETT consécutives réalisées entre janvier 2020 et décembre 2023 à Iqaluit et dans les collectivités environnantes. Les données relatives aux caractéristiques démographiques, aux résultats des examens échocardiographiques et aux décisions de prise en charge ont été recueillies et comparées entre patients autochtones et non autochtones. Les anomalies significatives étaient définies comme suit : valvulopathie modérée ou sévère, dysfonction de la valve prothétique, fraction d’éjection ventriculaire gauche (FEVG) < 50 %, anomalies régionales de la cinetique segmentaire (ACS), pression artérielle pulmonaire systolique ≥ 40 mmHg et cardiopathie congénitale.

**Résultats:**

Sur 662 patients (âge moyen : 55 ± 16 ans; 47 % de femmes), 530 (80 %) étaient autochtones. Des anomalies significatives ont été détectées chez 197 patients (30 %). Une valvulopathie modérée ou sévère était présente chez 67 patients (10 %), le plus souvent dégénérative (31 %), suivie d’étiologies fonctionnelles (25 %) et rhumatismales (16 %). Une FEVG réduite a été observée chez 16 % des patients, et des ACS chez 7 % des patients. Les mesures de prise en charge comprenaient un suivi localement (44 %), le transfert ou l’admission à l’hôpital (16 %) et une consultation à Ottawa (9 %). Comparativement aux patients non autochtones, les patients autochtones ont présenté des taux non ajustés plus élevés d’anomalies significatives (33 % *vs* 18 %; *p* < 0,01), de valvulopathie modérée ou sévère (12 % *vs* 5 %; *p* = 0,04) et de FEVG réduite ou d’ACS (21 % *vs* 13 %; *p* = 0,07).

**Conclusions:**

Les maladies cardiaques imposent un lourd fardeau aux patients de l’Arctique canadien, en particulier aux autochtones, comme en témoignent notamment les taux élevés de valvulopathie et de dysfonction ventriculaire. Une meilleure prévention et un meilleur accès aux soins cardiovasculaires sont essentiels pour réduire ces disparités.

Traditionally, Indigenous people presented with lower rates of cardiovascular diseases (CVDs) than non-Indigenous people, but in the past few decades, CVD incidence has risen markedly.^1^, ^2^, ^3^ The Indigenous population encounters distinct health challenges due to geographic isolation, limited access to healthcare services, and lower socioeconomic status. In comparison to the non-Indigenous Canadian population, Indigenous communities have a higher incidence of cardiovascular risk factors, experience more than double the prevalence of CVD, and face an increased risk of mortality in cases of acute coronary syndrome.^4^, ^5^, ^6^, ^7^ Although rheumatic heart disease (RHD) has been largely eradicated in Western countries, it persists as a preventable cause of heart failure and death in developing countries and among Indigenous people.^8^^,^^9^ Despite this evidence, contemporary data on echocardiographic features, particularly regarding valvular heart diseases (VHDs), remain scarce in this population.

For several years, physicians and sonographers from the University of Ottawa Heart Institute (UOHI) have been providing cardiology services (consultations and comprehensive transthoracic echocardiography [TTE]) in Iqaluit, Nunavut, the capital and largest city of the Nunavut Territory, and in several surrounding settlements. This study aims to report on the echocardiographic indications and findings, as well as on the management of all consecutive patients who underwent a clinically indicated comprehensive TTE through our program between 2020 and 2023.

## Methods

### Study design

Every 2-4 months, the UOHI sends a team composed of a senior cardiologist, a cardiology resident, and a sonographer to the Qikiqtani General Hospital in Iqaluit, Nunavut, the capital and largest city of the Nunavut Territory, for 1 or 2 weeks, depending on staffing resources and local needs. The cardiology team receives referrals from the Qikiqtani General Hospital, family physicians, and nurse practitioners located in Iqaluit as well as in remote locations. Although visits were initially less frequent, especially during the winter months, the program now operates year-round. Triage is conducted locally, with frequent communication between local healthcare providers and UOHI cardiologists, particularly when urgent investigations or treatments are required and when urgent transfer might be required. Echocardiograms are performed at the Qikiqtani General Hospital in Iqaluit, or at several Eastern Nunavut settlements, including Pangnirtung, Clyde River, Pond Inlet, Igloolik, Arctic Bay, and Qikiqtarjuaq. Patients from other communities (Kinngait, Grise Fiord, Sanirajak, and Kimmirut) are brought to Iqaluit for their visit and echocardiogram.

From our echocardiographic database, we identified all consecutive patients who underwent a clinically indicated comprehensive TTE during these missions between January 1, 2020 and December 31, 2023. If a patient had repeat TTE during the study period (2020-2023), the first echocardiogram was retained for this study. Clinical information, and indications for echocardiography and management were retrospectively extracted from our electronic medical record. Indigenous ethnicity (vs non-Indigenous) was determined if Inuktitut was recorded as the patient's first language, if the race was described as Indigenous in the medical record, or if the ethnicity was described as Indigenous in the consultation reports. The study was approved by our institutional review board. Individual patient consent was not required, as this was a retrospective analysis of existing data. The study was conducted under research license No. 01 016 25N-A, issued by the Nunavut Research Institute in accordance with the Nunavut’s Scientists Act.

### Clinical characteristics

Past medical history and medications were extracted when available. Dyspnea was classified according to the New York Heart Association (NYHA) classification, and angina was classified according to the Canadian Cardiovascular Society classification. Moderate or greater renal insufficiency was defined as a creatinine clearance of ≤ 60 mL/min/m^2^. Body surface area was calculated using the Dubois and Dubois formula.^10^ The rates of missing data in ethnicity or baseline characteristics are reported in the article text and tables.

### Echocardiography

All patients underwent a comprehensive 2-dimensional and Doppler TTE using a 5500 CV ultrasound machine (Philips Healthcare, Andover, MA) performed by a registered sonographer. All values were extracted from the original reports, and no measurements were retrospectively performed. The left ventricular ejection fraction (LVEF) was determined using the biplane Simpson’s method or visually; a 60% value was attributed when the LVEF was reported as normal. Mildly reduced LVEF was defined as an LVEF of 40%-49%, and moderate to severely reduced LVEF was defined as an LVEF of < 40%.^11^ Left atrial volume index was calculated using the area-length method and was considered to be enlarged at values above 34 mL/m^2^.^12^ Aortic stenosis (AS) was classified as mild, moderate, or severe, based on the mean pressure gradient (< 20 mm Hg, 20-39 mm Hg, and ≥ 40 mm Hg, respectively).^13^ Mitral valve anatomy and mitral regurgitation (MR) characteristics were assessed using multiparametric imaging, as were aortic (AR) and tricuspid regurgitation (TR).^14^ Moderate or severe mitral stenosis (MS) was defined by a mean pressure gradient ≥ 5 mm Hg and/or a valve area ≤ 1.5 cm^2^.^15^ VHD etiology was classified as rheumatic,^16^ degenerative (calcified aortic stenosis, calcified mitral valve stenosis, or regurgitation), myxomatous, functional (mitral and tricuspid), associated with aorta aneurysm or a bicuspid aortic valve, or congenital.^14^ Continuous wave Doppler measurement of the TR jet velocity was used in conjunction with the inferior vena cava collapsibility to calculate the systolic pulmonary artery pressure (sPAP). Pulmonary hypertension was defined as an sPAP ≥ 40 mm Hg.^17^ Significant echocardiographic abnormalities were defined as moderate or severe native VHD, moderate or severe prosthetic valve dysfunction, LVEF < 50% or left ventricular regional wall-motion abnormalities (RWMA), sPAP ≥ 40 mm Hg, or congenital heart disease.

### Statistical analysis

Continuous variables were expressed as the mean (± standard deviation) or median (25%-75%), and categorical variables were expressed as number (percent). Comparison between groups was performed using the Student *t* test, the Wilcoxon test, or the χ^2^ test, as appropriate. A *P* value < 0.05 was considered statistically significant. Statistical analyses were performed using JMP 17 software (SAS Institute, Cary, NC).

## Results

### Clinical characteristics

**Figure 1:**
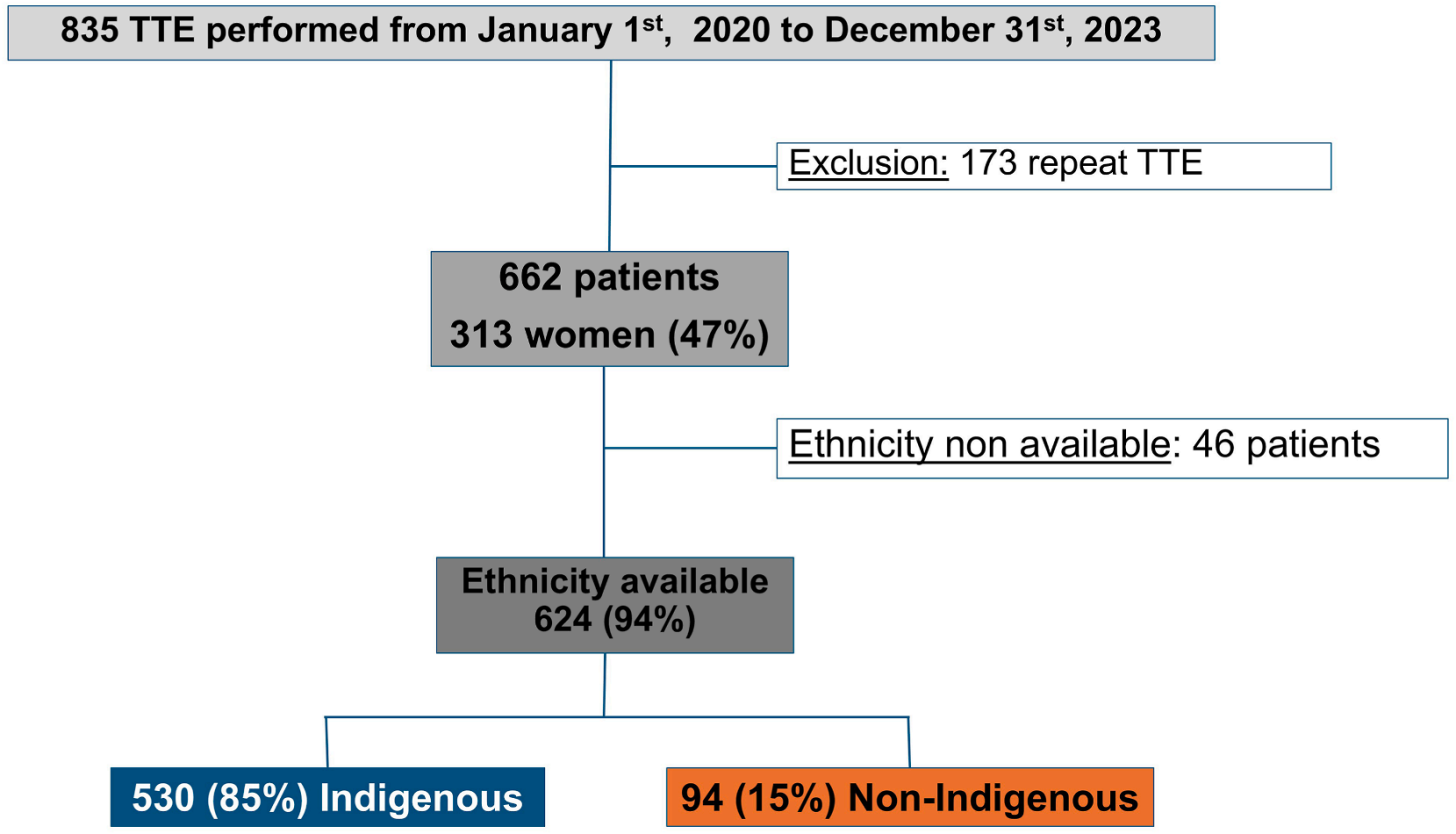
Flow chart of the study population. TTE, transthoracic echocardiogram.

Between January 2020 and December 2023, a total of 835 echocardiograms were performed in 662 patients (Fig. 1). The mean age was 55 ± 16 years, with 313 patients (47%) being women. Ethnicity data were available for 624 patients (94%), of whom 530 (85%) were identified as Indigenous. Clinical information was accessible in 566 patients (85%). Cardiovascular risk factors were common: 181 patients (32%) declared being active smokers, 260 (46%) were on treatment for hypertension, 193 (34%) were on treatment for dyslipidemia, and 111 (20%) were on treatment for diabetes mellitus. Among these patients, 58 (10%) had previously undergone cardiac surgery, including coronary artery bypass grafting in 20 patients (34%), aortic valve replacement in 15 patients (26%), and mitral valve replacement in 9 patients (16%). Fifty-three patients (9%) were in NYHA functional class III/IV, 99 patients (19%) had a history of heart failure, and 97 patients (17%) had Canadian Cardiovascular Society angina class > 1. Table 1 presents the baseline characteristics for the overall population.

**Table 1:** Baseline clinical characteristics of the study population

Characteristic	Overall∗ (N = 662)	Indigenous† N = 530	Non-Indigenous† N = 94	*p*	Indigenous female patients N = 265	Indigenous Male patients N = 265	*p*
Sex (female)	313 (47) 662	265 (50) 530	30 (32) 94	< 0.01			
Age, Y	55 ± 16 662	55 ± 17 530	55 ± 13 94	0.95	54 ± 17 265	57 ± 16 265	0.04
Height, cm	162 ± 12 659	160 ± 11 528	170 ± 11 94	< 0.01	154 ± 10 264	166 ± 8 264	< 0.01
Weight, kg	79 ± 24 660	77 ± 22 529	92 ± 27 94	< 0.01	71 ± 19 264	83 ± 24 265	< 0.01
Body surface area, m^2^	1.8± 0.3 659	1.8 ± 0.3 528	2.0 ± 0.3 94	< 0.01	1.7 ± 0.2 264	1.9 ± 0.3 264	< 0.01
Smoker Former Active	69 (12) 181 (32) 565	59 (13) 165 (36) 464	6 (7) 12 (14) 83	< 0.01	23 (10) 89 (37) 233	36 (16) 79 (34) 231	0.18
Hypercholesterolemia	193 (34) 564	151 (33) 462	35 (42) 84	0.11	65 (28) 231	86 (37) 231	0.04
Diabetes mellitus	111 (20) 566	79 (17) 464	27 (32) 84	< 0.01	27 (12) 231	52 (22) 233	< 0.01
Hypertension	260 (46) 565	211 (46) 463	42 (50) 84	0.45	99 (43) 231	112 (48) 232	0.24
Prior acute coronary syndrome / myocardial infarction	39 (7) 564	33 (7) 462	5 (6) 84	0.69	11 (5) 231	22 (10) 231	0.04
Transient ischemic attack / stroke	69 (12) 564	58 (13) 463	6 (7) 83	0.17	23 (10) 232	35 (15) 231	0.09
Peripheral arterial disease / abdominal aortic aneurysm	9 (2) 563	8 (2) 462	0 (0) 83	0.22	2 (1) 231	6 (3) 231	0.15
Moderate or severe renal insufficiency	30 (5) 662	27 (5) 530	2 (2) 94	0.17	8 (3) 265	19 (7) 265	0.03
Pacemaker	32 (6) 564	26 (6) 463	4 (5) 83	0.77	14 (6) 232	12 (5) 231	0.69
History of atrial fibrillation	62 (11) 564	43 (9) 463	18 (22) 83	< 0.01	20 (9) 232	23 (10) 231	0.62
Chronic pulmonary disease	111 (20) 562	93 (20) 462	15 (19) 83	0.66	28 (12) 231	65 (28) 231	< 0.01
History of infective endocarditis	3 (0.5) 562	3 (1) 461	0 (0) 83	0.46	1 (0.4) 230	2 (1) 231	0.56
History of cardiac surgery	58 (10) 561	48 (10) 461	8 (10) 82	0.85	23 (10) 231	25 (11) 230	0.75
New York Heart Association class III/IV	53 (9) 564	42 (9) 463	9 (11) 83	0.61	17 (7) 231	25 (11) 232	0.20
History of congestive heart failure	99 (19) 564	90 (19) 463	9 (11) 83	0.04	43 (19) 231	47 (20) 232	0.65
Canadian Cardiovascular Society angina ≥ 1	97 (17) 561	78 (17) 463	16 (20) 83	0.59	29 (13) 231	49 (21) 232	0.01

Data are presented as number (%), or mean ± standard deviation, unless otherwise indicated followed by number of patients with valaibale information.

∗ Clinical information available in 566 patients.

† Ethnicity was obtained in 624 patients.

### Echocardiographic characteristics

**Figure 2:**
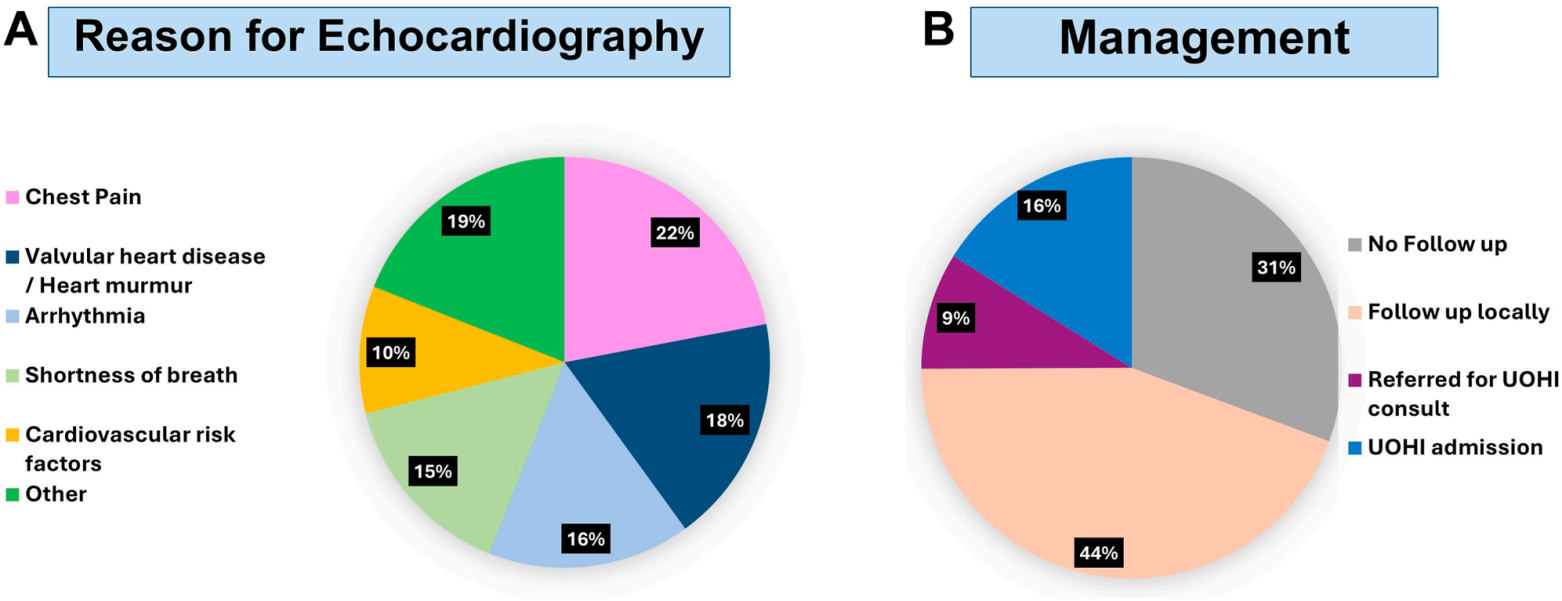
(**A**) Main reason for echocardiography in the overall population and (**B**) management following echocardiography and clinical assessment. UOHI, University of Ottawa Heart Institute.

The primary reason for echocardiography included chest pain in 147 patients (22%), heart murmur or follow-up of known VHD in 119 patients (18%), arrhythmia in 103 patients (16%), shortness of breath in 100 patients (15%), and cardiovascular risk factors in 68 patients (10%; Fig. 2A).

The mean ejection fraction was 56% ± 10%; 41 patients (6%) had mildly reduced LVEF (40%-49%), and 61 patients (10%) had moderately or severely reduced LVEF (< 40%). RWMAs were noted in 48 patients (7%). The mean sPAP was 27 ± 10 mm Hg, and 33 patients (11%) had an sPAP ≥ 40 mm Hg. Of note, sPAP was not measurable in 294 cases (44%), due to the absence of TR. Moderate or severe VHD was noted in 67 patients (10%). MR was noted in 22 patients (33%), AS in 17 patients (25%), AR in 16 patients (24%), TR in 15 patients (22%), and MS in 7 patients (10%). Eleven patients (16%) had multiple moderate or severe native VHD. The etiology of VHD was degenerative in 21 patients (31%), functional in 17 patients (25%), RHD in 11 patients (16%), associated with an aortic aneurysm or bicuspid aortic valve in 12 patients (18%), and related to myxomatous valve prolapse in 5 patients (7%). Notably, an additional 14 patients (2%) had previously undergone valvular surgery for RHD; thus, overall, 25 patients (4%) had current or prior significant rheumatic VHD. Prosthetic valve dysfunction was reported in 6 patients (1%). Twelve patients (2%) had adult congenital heart disease (4 patients had corrections in childhood [eg, tetralogy of Fallot], and 8 remained uncorrected—7 ventricular septal defects and 1 pulmonary stenosis). A summary of the echocardiographic characteristics of the population is provided in Table 2. Overall, significant echocardiographic abnormalities were identified in 197 patients (30%; Central Illustration).

**Table 2:** Echocardiographic characteristics of the study population

Characteristic	Overall (N = 662)	Indigenous∗ N = 530	Non-Indigeneous∗ N = 94	*p*	Indigenous female N = 265	Indigenous male N = 265	*p*
Reason for echocardiography				< 0.01			< 0.01
Chest pain	147 (22)	110 (21)	28 (30)		38 (14)	72 (27)	
Valvular heart disease	119 (18)	100 (19)	13 (14)		60 (23)	40 (15)	
Arrhythmia	103 (16)	75 (14)	21 (22)		39 (15)	36 (14)	
Shortness of breath	100 (15)	92 (17)	5 (5)		47 (18)	45 (17)	
Cardiovascular risk factors	68 (10)	52 (10)	7 (7)		28 (11)	24 (9)	
Other	125 (19)	101 (19)	20 (21)		53 (20)	48 (18)	
Significant echocardiographic abnormalities†	197 (30) 662	176 (33) 530	17 (18) 94	< 0.01	74 (28) 265	102 (38) 265	< 0.01
Left ventricular ejection fraction, % < 40 40–49 ≥ 50	56 ±10 61 (10) 41 (6) 539 (84) 641	56 ± 11 54 (10) 36 (7) 425 (83) 515	58 ± 8 6 (7) 3 (3) 81 (90) 90	0.08 0.19	57 ± 9.3 19 (7) 12 (5) 225 (88) 256	53 ± 11 35 (14) 24 (9) 200 (77) 259	< 0.01 < 0.01
Regional wall-motion abnormalities	48 (7) 662	37 (7) 530	10 (11) 94	0.23	8 (3) 265	29 (11) 265	< 0.01
Reduced ejection fraction or regional wall-motion abnormalities	127 (20) 645	111 (21) 519	12 (13) 90	0.07	36 (14) 258	75 (29) 261	< 0.01
Left atrium, mL/m^2^	36 ± 17 629	37 ± 18 506	31 ± 11 87	< 0.01	38 ± 19 256	36 ± 16 250	0.38
Systolic pulmonary arterial pressure, mm Hg	27 ± 10 368	28 ± 10 239	26 ± 11 44	0.28	28 ± 10 135	28 ± 10 104	0.88
Systolic pulmonary arterial pressure ≥ 40 mm Hg	33 (11) 368	31 (13) 239	2 (5) 44	0.11	16 (12) 135	15 (14) 104	0.56
Moderate or severe native valvular heart disease	67 (10) 662	62 (12) 530	5 (5) 94	0.04	34 (13) 265	28 (11) 265	0.41
Current or prior rheumatic heart disease	25 (4) 662	25 (5) 530	0 (0) 94	0.03	17 (6) 265	8 (3) 265	0.04
Congenital heart disease	12 (2) 662	12 (2) 530	0 (0) 94	0.04	6 (2) 265	6 (2) 265	0.99

Data are presented as number (%), or mean ± standard deviation, unless otherwise indicated followed by number of patients with valaibale information.

∗ Ethnicity was obtained in 624 patients.

† Significant echocardiographic abnormalities were defined as moderate or severe native valvular heart diseases, moderate or severe prosthetic valve dysfunction, left ventricular ejection fraction < 50% or left ventricular regional wall-motion abnormalities, systolic pulmonary arterial pressure ≥ 40 mm Hg, or congenital heart disease.

**Central Illustration:**
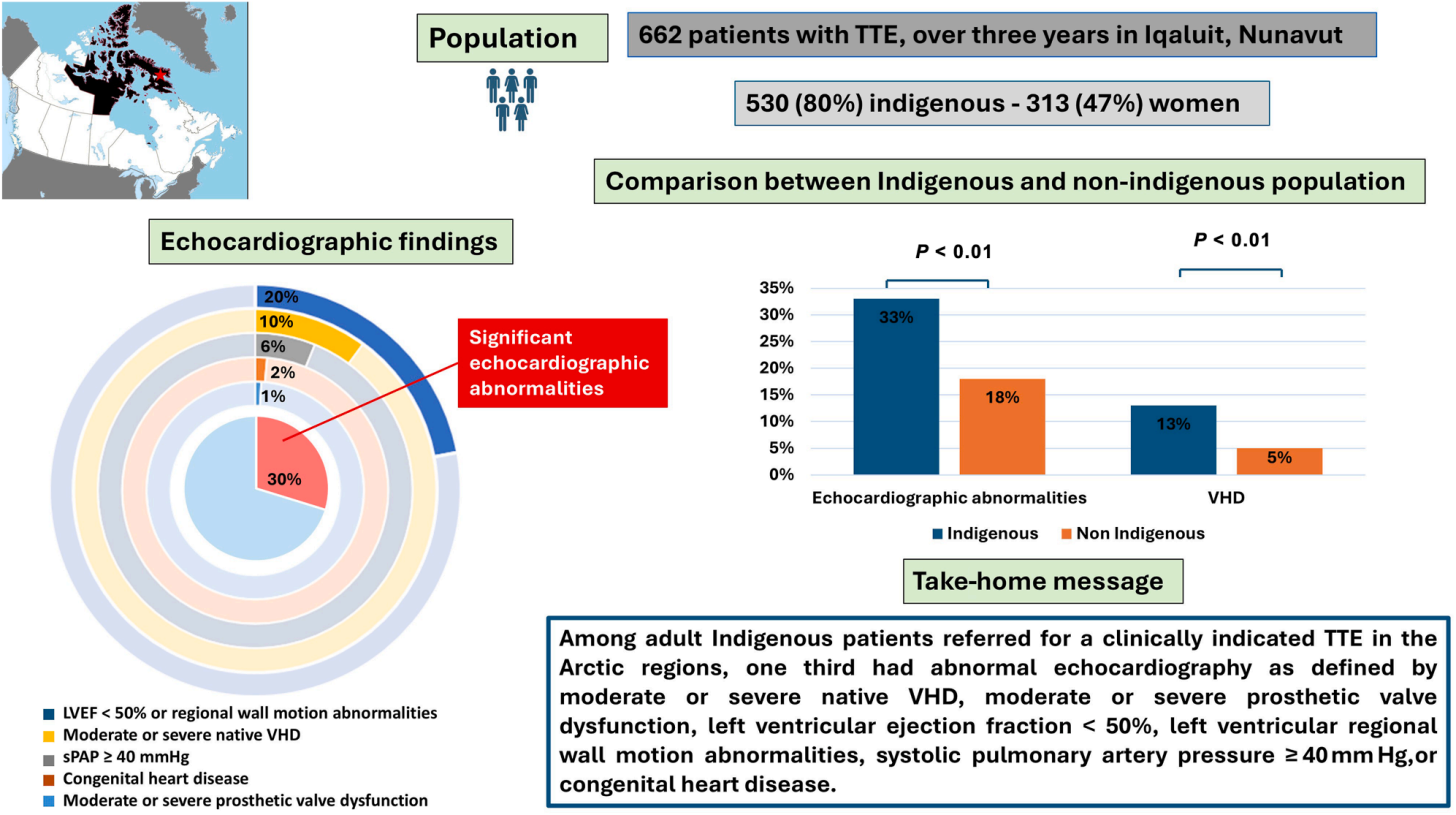
Echocardiographic findings and differences between Indigenous and non-Indigenous individuals. ACHD, adult congenital heart disease; EF, ejection fraction; LV, left ventricular; sPAP, systolic pulmonary arterial pressure; TTE, transthoracic echocardiogram; VHD, valvular heart disease.

### Management

No follow-up was deemed necessary for 198 patients (31%). In 286 patients (44%), a follow-up visit was scheduled locally with either a cardiologist or general practitioner. A total of 58 patients (9%) were referred for a consultation at the UOHI, and an additional 104 patients (16%) were recommended for transfer and admission at the UOHI (Fig. 2B). The primary reasons for UOHI admission included need for an angiogram in 23 patients (22%), consideration for cardiac surgery in 12 patients (12%; 7 for valvular surgery and 5 for coronary artery bypass grafting), and further medical management in 69 patients (66%). Among those admitted for medical management, 26 patients (38%) were evaluated for chest pain and/or angina, 18 (26%) for heart failure, 10 (14%) for further assessment of VHD, 8 (12%) for arrhythmia, and 7 (10%) for other reasons.

### Indigenous subset

#### Clinical presentation

Although age was not different (55 ± 17 years in the Indigenous group vs 55 ± 13 years in the non-Indigenous group, *P* = 0.95), the proportion of women was significantly higher in the Indigenous group (265 [50%] vs 30 [32%], respectively, *P* < 0.01). Compared to the non-Indigenous group, the Indigenous group showed a significantly higher rate of active smoking (165 patients [36%] vs 12 patients (14%), respectively, *P* < 0.01), but a lower rate of diabetes mellitus (79 patients [17%] vs 27 patients [32%], respectively, *P* < 0.01). Additionally, the Indigenous group had a lower prevalence of atrial fibrillation (43 patients [9%] vs 18 patients [22%], respectively, *P* < 0.01).

#### Echocardiographic indications and findings

**Figure 3:**
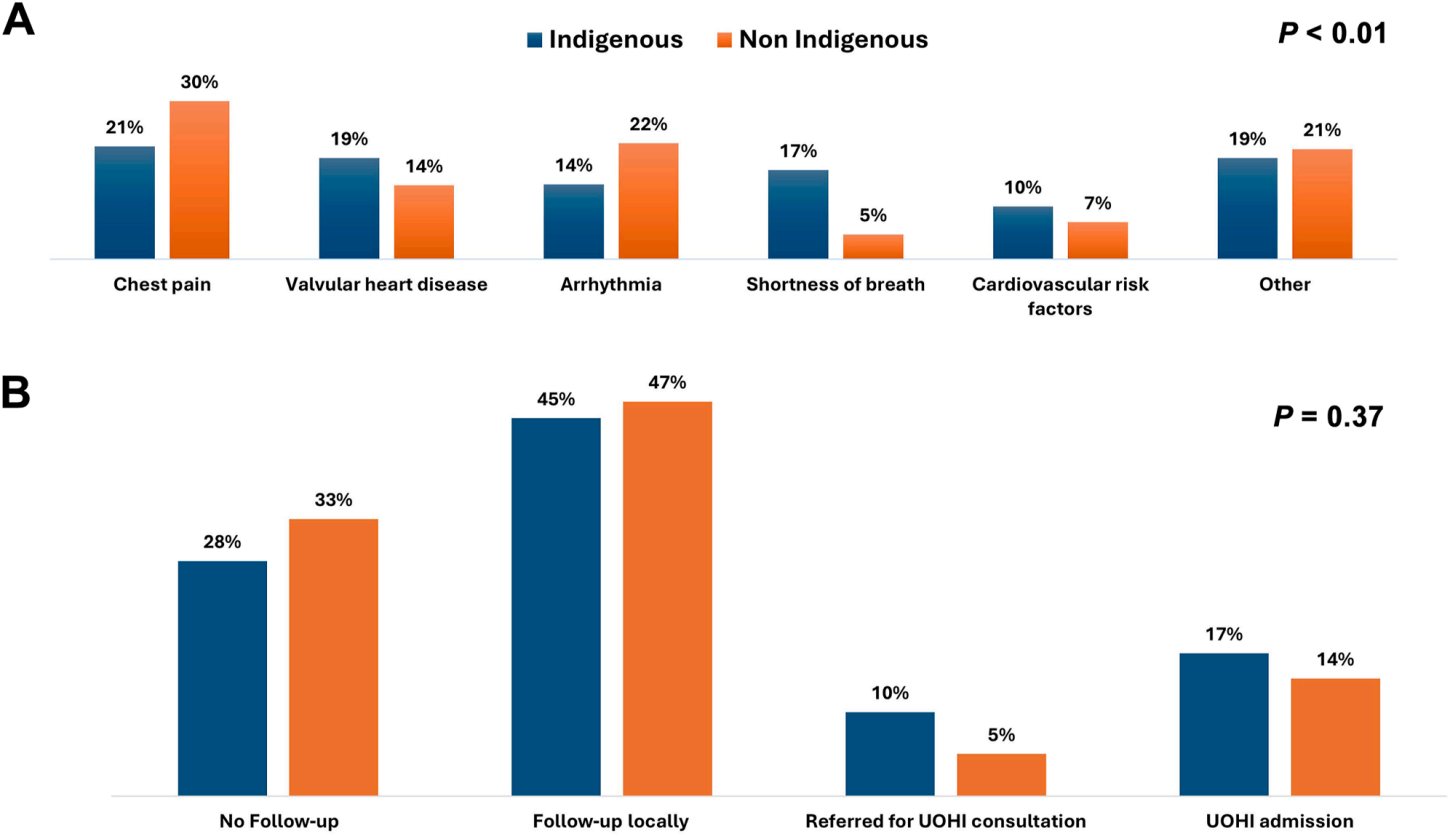
Comparison between Indigenous and non-Indigenous patients in regard to (**A**) reason for echocardiography and (**B**) management of patients. UOHI, University of Ottawa Heart Institute.

Compared to the non-Indigenous group, the reason for echocardiography in the Indigenous group was more often shortness of breath (92 patients [17%] vs 5 patients [5%], respectively, *P* < 0.01), and less frequently chest pain (110 patients [21%] vs 28 patients [30%], respectively, *P* < 0.01; Fig. 3A). Significant echocardiographic abnormalities were identified in 176 patients (33%) in the Indigenous group, compared to 17 patients (18%) in the non-Indigenous group (*P* < 0.01). Moderate or severe native VHD was observed in 62 patients (12%) in the Indigenous group, but in only 5 patients (5%) in the non-Indigenous group (*P* = 0.04). RHD was reported in 25 (5%) of the Indigenous group and none of the non-Indigenous group (*P* < 0.01). Additionally, all 12 patients (2%) with congenital heart disease were in the Indigenous group (*P* = 0.04). A strong trend toward a higher prevalence of moderate or severely reduced LVEF or RWMA also was observed between the Indigenous and non-Indigenous groups (111 patients [21%] vs 12 patients [13%], respectively, *P* = 0.07; Table 2).

#### Management

The overall rates of patients requiring no follow-up, local follow-up, or follow-up and admission at our institution were not significantly different between the Indigenous and non-Indigenous patients (*P* = 0.37; Fig. 3B). However, a trend suggested a higher need for consultation or transfer for admission to our institution among Indigenous patients (27% vs 19%, *P* =0 .12).

#### Sex differences

In the Indigenous group, significant echocardiographic abnormalities were more often observed in the male patients than in the female patients (102 patients [39%] vs 74 patients [28%], respectively, *P* < 0.01). Male patients also presented more frequently with LVEF < 50% (23% vs 12%, respectively, *P* < 0.01). Moderate or severe VHD was reported in 34 female patients (13%) and 28 male patients (11%; *P* = 0.41). Female patients were more frequently diagnosed with MS (*P* = 0.04) and had more RHD overall compared to male patients (17 [6%] vs 8 (3%), respectively, *P* = 0.04). The rate of referral for a consultation or an admission to our institution was not different between women and men (24% vs 29%, respectively, *P* = 0.16).

## Discussion

In this series of consecutive patients referred for a TTE in the Arctic region of Nunavut, the main results can be summarized as follows: (i) significant echocardiographic abnormalities were reported in one third of patients; (ii) compared to the non-Indigenous group, Indigenous patients had an almost 2-fold increased prevalence of significant echocardiographic findings, including VHD; and (iii) among Indigenous patients, women present less frequently with significant echocardiographic abnormalities, but they have a higher frequency of RHD and MS compared to that in men. The rates of referral for consultation or transfer for admission at our institution were relatively high and were similar in female and male Indigenous patients.

### Clinical presentation

In this cohort population of patients referred for an echocardiography, the mean age was 55 years, and 47% were women. Nearly 10% of participants experienced NYHA class III or IV dyspnea, and angina was reported in nearly 1 in 5 patients. We observed a lower rate of diabetes in the Indigenous group than in the non-Indigenous group. Based on the Canadian Community Health Survey, in 2017, the prevalence of diabetes mellitus in Inuit patients was estimated to be around 1%,^18^ compared to 8.8% in the general Ontario population.^19^ Smoking rates in the present study were substantially lower than those reported in prior Territorial surveys (up to 75% in Nunavut).^20^ Although Indigenous individuals were 2.5 times more likely to smoke compared to non-Indigenous individuals (36% vs 14%), smoking rates might have been underreported in the present study. Interestingly, despite a higher frequency of enlarged left atrium, and a high prevalence of cardiovascular risk factors, the Indigenous group exhibited a lower rate of atrial fibrillation. This phenomenon has been described as a "racial paradox"—that is, populations with high risk factors, such as those with Indo-Asian ancestry, have lower rates of atrial fibrillation than expected. However, this result has not been confirmed previously in Canadian Indigenous populations, warranting further investigation.^21^

### Echocardiographic findings

Approximately 10% of the cohort presented with moderate or severe native VHD. A study conducted in Olmsted County, Minnesota, reported a prevalence of VHD of 1.8% in patients referred for a clinically indicated echocardiographic evaluation.^22^ Systematic population screening studies have provided further insights into the burden of VHD.^23^ The OxValve study, a prospective cohort study conducted in the United Kingdom between 2007 and 2016, found a prevalence of clinically significant VHD of 2.4% in individuals aged 60 years or older, with 2.2% having moderate VHD and 0.2% severe VHD.^24^ The relatively young age of our cohort (mean age, 55 years) underscores a disproportionately higher burden and earlier onset of VHD compared to the findings in these studies, suggesting regional or demographic variations. In our cohort, the 3 main moderate or severe VHDs were MR, AS, and AR, not different than previously reported in other populations.^24^

Among patients with VHD, the 3 main etiologies were degenerative in 31% of patients, functional in 25%, and rheumatic in 16%. Although RHD remains a major health concern in the Arctic region, degenerative and functional etiologies have become more prevalent, reflecting a double burden. On the one hand, the Indigenous population still experiences a higher rate of RHD, driven by low-level socioeconomic conditions, geographic isolation, and access to care, including timely access to primary and preventive healthcare. Together, these factors contribute to delayed diagnosis and suboptimal prevention of acute rheumatic fever, ultimately increasing the risk and severity of RHD.^25^ In rural and remote Indigenous communities in Northwestern Ontario, invasive Group A Streptococcus infections occur at a rate 10 times higher than the provincial and national averages,^8^ and the incidence of rheumatic fever is 21.3 cases per 100,000, or 75 times higher than the national average.^26^ According to a global survey on RHD conducted between 1990 and 2015, the estimated prevalence of RHD in 2015 was 4.4% in endemic countries and 0.03% in non-endemic countries. In our cohort of patients undergoing echocardiography, 3.8% of patients (25 of 662) had moderate or severe RHD. Although this number cannot be extrapolated to the Nunavut population, as this was not a systematic screening study,^27^ a relevant comparison can be made with a retrospective, multicentre study conducted in 2 echocardiography laboratories in Uganda, where 9.8% of the cohort received an echocardiographic diagnosis of RHD.^28^ On the other hand, the Indigenous group also incurs a high rate of degenerative VHD, fueled by industrialization-related risk factors, such as tobacco smoking and physical inactivity.^1^ This epidemiologic transition highlights the need for targeted public health interventions to address both RHD and degenerative heart conditions in this population. Additionally, almost 20% of our cohort that was referred for TTE presented with a reduced LVEF or RWMA. By comparison, an international cohort study conducted between Russia and Norway reported that 12% of patients presented with an LVEF < 50%,^29^ and the Framingham cohort reported a rate of left ventricular dysfunction of only 5.8% between 2005 and 2014.^30^ Finally, in our cohort, 2% presented with adult congenital heart disease. A higher frequency of grownup congenital heart disease, including conditions such as Tetralogy of Fallot and ventricular septal defects has been reported previously in the Indigenous group, compared to that in the non-Indigenous group.^31^ To account for this high prevalence, factors such as genetic predisposition, a diet low in folates and vitamin A, and periconceptional alcohol use have been suggested. Once again, this study was not a systematic screening study and does not allow us to derive prevalence in Nunavut. For reference, the overall prevalence of adult congenital heart disease in the US is 1 in 150 patients (0.7%).^32^

Critically, Indigenous identity is not intrinsically associated with an increased risk of CVD; rather, the elevated burden observed in this study likely reflects the impact of social determinants of health, geographic isolation, and limited access to primary care, preventive services, and cardiology expertise. The high prevalence of cardiovascular and valvular disease should therefore be interpreted in the context of a referred population with a high pretest probability of disease, not as evidence of a biological predisposition linked to Indigenous status. Similar disease patterns would be expected in any isolated or underserved population facing barriers to prevention, delayed diagnosis, and limited treatment of cardiovascular risk factors.

### Sex differences

In our study, as expected, male patients exhibited more frequent risk factors, such as diabetes, and a higher incidence of coronary artery disease and chronic pulmonary disease, as compared to female patients. Male patients also were referred more frequently for chest pain than were female patients. Significant echocardiographic abnormalities were identified more commonly in men than women. Males had a higher prevalence of reduced LVEF, likely linked to the high frequency of coronary artery disease.^33^ Clinically, this finding aligns with the recognized pattern of men presenting more often with heart failure with reduced ejection fraction, and women more frequently developing heart failure with preserved ejection fraction.^34^ Conversely, our female patients had a higher prevalence of RHD and MS compared to male patients, as has been observed in other populations.^35^, ^36^, ^37^ These results highlight the fact that VHD should be a particular focus in women.^38^

### Limitations

Several limitations deserve to be discussed. First, this study was not a population-based study with systematic screening but rather a selected population referred for echocardiography evaluation. All patients underwent clinically indicated echocardiography following referral to a travelling cardiology clinic, resulting in a selected population with a high pretest probability of CVD. Accordingly, the generalizability of these findings to the broader community should be interpreted with caution. However, the high burden of disease observed in our population, which exceeds the prevalences typically observed in an echocardiography laboratory, highlights an important unmet need in the identification of disease in the Arctic population.^22^ In addition, the Nunavut population was 36,858 inhabitants in 2021, and we collected 662 patients referred for a clinically indicated TTE over 4 years, which represents 2% of the population; this rate corresponds to more than 700,000 of the entire Canadian population.^39^

Second, the ethnicity or first language was not recorded systematically in our electronic medical system, resulting in missing data, but only in 6% of the patients. Similarly, clinical information was extracted from chart reviews and could not be obtained in all patients. Third, we cannot exclude the possibility that some patients with known cardiovascular abnormalities may have had a prior TTE before 2020 and were referred for a follow-up TTE during the study period that did not reflect a new finding. Fourth, our non-Indigenous comparator group primarily consists of men, likely temporary workers, who may be healthier than the general Canadian population. However, despite the potential for selection bias, an important point to note is that the non-Indigenous patients, like the Indigenous patients, were referred for clinically indicated echocardiograms. In addition, the magnitude of the differences observed, supported by a review of the literature, strongly suggests that these findings are genuine. Nevertheless, a cautious interpretation remains necessary. Fifth, the management strategies described in this study were not solely based on echocardiographic findings but were integrated with comprehensive clinical evaluations. Sixth, all comparisons between Indigenous and non-Indigenous patients were unadjusted. Consequently, the observed differences in echocardiographic findings may be influenced by baseline clinical characteristics, comorbidities, or referral patterns, and should therefore be interpreted with caution. However, higher rates of echocardiographic abnormalities persisted after adjustment for age and sex (odds ratio = 2.48, 95% confidence interval [1.45-4.49], *P* = 0.002). In addition, the non-Indigenous group was composed predominantly of men, likely representing temporary workers who may have been comparatively healthier, potentially introducing a selection bias in the comparison. Of note, the duration of residence in Nunavut among non-Indigenous participants was not available. Seventh, sPAP could not be measured in 44% of cases because TR was absent. As pulmonary hypertension may still be present in the absence of measurable TR, the true prevalence of pulmonary hypertension may have been underestimated. As assessment relied on primarily echocardiography, no right heart catheterization data were available in this cohort. Finally, in Nunavut, the Indigenous population is comprised predominantly of Inuit patients, who report primarily Inuktitut as their first language. Therefore, the results of this study may not apply in other First Nations communities.

## Conclusion

In this retrospective analysis of patients referred for TTE through a travelling cardiology clinic in Canada’s North, we observed a high prevalence of significant echocardiographic abnormalities, including VHD. These findings underscore the substantial burden of CVD among patients requiring specialized cardiac assessment and care. Within this referred cohort, RHD remains a significant concern in Nunavut, and degenerative valvular disease has emerged as the predominant etiology of VHD. Although these results should be extrapolated to the general population with caution, they highlight the complex and evolving cardiovascular care needs of individuals living in remote and underserved communities. They also emphasize the importance of improving access to timely cardiac evaluation, implementing targeted screening strategies for high-risk populations, and strengthening primary prevention efforts tailored to resource-limited settings.
